# Funding for kidney transplantation

**DOI:** 10.1590/2175-8239-JBN-2021-E007

**Published:** 2021-06-18

**Authors:** Valter Duro Garcia, Elizete Keitel

**Affiliations:** 1 Santa Casa de Misericórdia de Porto Alegre, Centro de Nefrologia e Transplante Renal, Porto Alegre, RS, Brasil.; 2 Universidade Federal de Ciências da Saúde de Porto Alegre, Porto Alegre, RS, Brasil.

Kidney transplantation, a precursor of other organ transplants, started in Brazil in the mid-1960s due to the determination and pioneering spirit of nephrologists, urologists, and general surgeons, without the participation of government agencies for control or financing. This heroic phase lasted until 1987, when the Ministry of Health, initially through the SIRC-Trans program (Integrated System for Chronic and Transplanted Renal Patients) and later in 1993 through the SIPAC-Rim program (Integrated System of Highly Complex Patients), started to control and partially finance kidney transplants. After this idealistic phase, we entered in 1998 the professional phase of transplants, with the establishment of a transplant policy in the country. In this phase, an organizational model was created based on the Spanish Model (National System, National Central, State Centrals, and Hospital Transplant Coordinators), and an adequate funding was established for the time for all stages of transplants through the Strategic Actions Fund and Compensation (FAEC)^1^^,^ 2.

In 1998, the reimbursement of kidney transplantations with deceased donors had a cost 18% higher than transplantation with living donor (R$ 10,013.00 and 8,473.24 respectively), and with readjustments this difference became 30% in 2012 (R$ 27,622.67 and R$ 21,238.82, respectively). In that year, a financial increase (IFTDO) of 30 to 60% was also granted for some of the donation and transplant procedures, including kidney transplantation, according to the number, modality, and complexity of the transplants performed^2^. Since then, there has been no readjustment in kidney transplant funding values.

There are few studies in Brazil analyzing the costs of kidney transplantation, with the majority comparing dialysis treatment and all studies demonstrating that the transplant has the best cost-benefit ratio^3^^-^ 5. In a study with 80 transplants performed at the Santa Casa de Porto Alegre, between 2012 and 2013, the average cost of kidney transplantation with deceased donor was R$ 30,094.86 and with live donor R$ 20,004.76, but there was no more detailed analysis of transplants such as separation by standard deceased donor or with expanded criteria donor^6^.

In a well-designed study conducted at HC-USP, Quinino et al. (2021)^(7 )^compared the costs of kidney transplants with deceased donor in recipients with rapid (Group 1) or slow creatinine decrease without the need for dialysis (Group 2) and with the need for dialysis in the first week (Group 3). The authors demonstrated that the costs ​​were significantly higher in groups 2 (36%) and 3 (166%) when compared with group 1. Based on these results, they suggest asking health authorities to reimburse kidney transplants in non-sensitized recipients with different values ​​for these three groups^7^.

It seems fully justifiable to claim different values ​​for kidney transplants with greater complexity or morbidity. Instead of proposing an increase in values ​​depending on an outcome (percentage of creatinine decrease or need for dialysis), the proposition of differentiated payment by risk factors seems to be more viable, as already occurs in kidney transplantation with a living and deceased donor.

An alternative and broader way to be proposed to health authorities is the previously established differentiated funding (30 to 50% higher) for more complex groups of kidney transplant recipients with deceased donor, such as:

- pediatric recipients aged 12 years or less, as already occurs in hemodialysis funding in this population^8^;
- kidney recipients from higher risk donors (KDPI> 85% or other criteria to be adopted);
- recipients with high immunological risk (presence of DSA> 2,000 and other situations to be discussed).

The use of renal perfusion machines discussed by Quinino et al.^7^ deserves a more in-depth analysis of its real value in Brazil. There are studies in our country showing a lower incidence and duration of delayed graft function and a consequent shorter hospital stay^9^^,^ 10 with the use of perfusion machines, however the cost is high. Unlike other organs, the kidney is perfused before allocation, and hospitals in each State must have an evaluation of this approach. The State should be responsible for the purchase of the machines, disposable material and perfusion, if that is the protocol of choice. In this case, the indications and the result control must be evaluated by the State Kidney Technical Chamber. This could prevent what happened in some States, in which there was the acquisition of perfusion machines that were used for a period of months or a few years, having their use suspended due to the high cost of the disposable material and because the results could not be evaluated, since the indications for the use of continuous perfusion machine were not properly defined.

The analysis of the kidney transplantation financing, as well as of the financing of other organs’ transplantation, after more than 20 years in this professional phase, shows that there is still no reimbursement for some fundamental laboratory tests for the monitoring of transplanted patients, such as quantitative polymerase chain reaction test kits for CMV, EBV, and BKV, which allow for the early diagnosis of these diseases. Also, there is no provision of effective oral medication for the treatment of the CMV disease. These simple measures are cost-effective, as they reduce morbidity, mortality, and length of stay.

In addition, in a broader analysis of transplants’ financing, the costs ​​of immunological investigation and post-transplant follow-up should be reviewed as funding has not been updated for more than 20 years^2^.

The article by Quinino et al. at HC-USP analyzes the costs of hospitalization for kidney transplantation with a large sample of patients and proposes changes in the reimbursement process. We await further studies proposing changes that can improve the financing system for transplants in Brazil.
